# IoT in Healthcare: Achieving Interoperability of High-Quality Data Acquired by IoT Medical Devices

**DOI:** 10.3390/s19091978

**Published:** 2019-04-27

**Authors:** Argyro Mavrogiorgou, Athanasios Kiourtis, Konstantinos Perakis, Stamatios Pitsios, Dimosthenis Kyriazis

**Affiliations:** 1 Department of Digital Systems, University of Piraeus, M. Karaoli & A. Dimitriou 80, 18534 Piraeus, Greece; kiourtis@unipi.gr (A.K.); dimos@unipi.gr (D.K.); 2 Singular Logic EU Projects Department, Achaias 3, 14564 Kifisia, Greece; kperakis@ep.singularlogic.eu (K.P.); stamatis.pit@gmail.com (S.P.)

**Keywords:** internet of things, healthcare, medical devices, heterogeneous devices, data heterogeneity, quality assessment, data cleaning, data quality, data interoperability

## Abstract

It is an undeniable fact that Internet of Things (IoT) technologies have become a milestone advancement in the digital healthcare domain, since the number of IoT medical devices is grown exponentially, and it is now anticipated that by 2020 there will be over 161 million of them connected worldwide. Therefore, in an era of continuous growth, IoT healthcare faces various challenges, such as the collection, the quality estimation, as well as the interpretation and the harmonization of the data that derive from the existing huge amounts of heterogeneous IoT medical devices. Even though various approaches have been developed so far for solving each one of these challenges, none of these proposes a holistic approach for successfully achieving data interoperability between high-quality data that derive from heterogeneous devices. For that reason, in this manuscript a mechanism is produced for effectively addressing the intersection of these challenges. Through this mechanism, initially, the collection of the different devices’ datasets occurs, followed by the cleaning of them. In sequel, the produced cleaning results are used in order to capture the levels of the overall data quality of each dataset, in combination with the measurements of the availability of each device that produced each dataset, and the reliability of it. Consequently, only the high-quality data is kept and translated into a common format, being able to be used for further utilization. The proposed mechanism is evaluated through a specific scenario, producing reliable results, achieving data interoperability of 100% accuracy, and data quality of more than 90% accuracy.

## 1. Introduction

Internet of Things (IoT) technologies are increasing rapidly, having become a milestone advancement in the digital healthcare domain [1,2,3]. According to [4], today 50 million medical devices are in use, and it is anticipated that by 2020 there will be over 161 million of them connected worldwide. This huge expansion of the IoT medical devices market is due to the evolution of high-speed networking technologies, and the increasing adoption of wearable devices, smartphones, and other mobile platforms in healthcare [5]. Therefore, nowadays there exists a large amount of IoT medical devices, which is gradually increasing with every passing day, resulting into a myriad of heterogeneous devices that are connected to the healthcare IoT world.

However, these devices are typically characterized by a high degree of heterogeneity [6], producing huge amounts of health and fitness data in heterogeneous formats [7]. Hundreds of healthcare organizations deal everyday with challenges in extracting data from different kinds of medical devices, affecting both patient care and medical research [8]. Nevertheless, all these healthcare organizations are facing many difficulties in managing all these huge amounts of data, mainly lacking an integrated data exchange system [9]. In order to exchange data with as many organizations as possible, interoperability is the only way for letting systems interact with each other [10], being considered as a necessity in the electronic healthcare systems for resolving data heterogeneity issues.

However, all this exchanged medical data may be not only of heterogeneous format but also of different levels of quality [11]. Henceforth, even if all this data will be interoperable among each other, it is not sufficient to transform all this data into a common format, since all of it is extremely crucial as it drives medical decision making [12]. On the contrary, it would be wiser and more effective to take into consideration the different levels of quality that this data may have, thus transforming only the data that is of a high-quality level. Therefore, the problem that arises is on the one hand the necessity of the heterogeneous devices’ derived data to be fully interoperable, and on the other hand the difficulty of identifying the quality of this enormous amount of data. For that reason, it is of crucial importance not only to find out an automated way for making these devices’ derived data interoperable, but also to find out an automated way for measuring the quality of all this data so as to apply the interoperability transformations only upon this data. However, the authors of many current studies tried to address the one aspect of the aforementioned challenges (i.e., data quality) [13,14,15,16], whereas some others tried to address the other main aspect of these challenges (i.e., data interoperability) [17,18,19,20]. Henceforth, until today, all the developed researches have tried to solve separately the challenges of data quality and data interoperability, neglecting to propose a holistic approach for successfully achieving data interoperability upon high-quality data that derive from heterogeneous devices.

To address this challenge, in this manuscript a mechanism is proposed for gathering heterogeneous IoT medical devices’ data, automatically extracting the data that is of high-quality and making it interoperable. Based on this mechanism, initially all the available heterogeneous IoT medical devices are discovered and connected into the mechanism, which is responsible for collecting their data. Once these devices are connected and their data is successfully gathered, the cleaning of it takes place, from which the results of each device’s dataset cleaning derive. The latter are combined with the corresponding overall data quality measurements that are captured from each different device in combination with its derived data, so as to decide whether the connected devices’ derived data will be considered as reliable or not. Consequently, only the reliable data is kept and translated into an interoperable format, and thus are kept to be used for further analysis. The proposed mechanism is evaluated through a specific use case, by gathering data from heterogeneous IoT medical devices, in order to clean it and capture its quality levels, and finally make interoperable only the cleaned data that are of high-quality.

This manuscript is organized as follows: Section 2 (Materials and Methods) analyzes the proposed mechanism for achieving data quality assessment and interoperability transformation among different kinds of medical data that derive from heterogeneous IoT medical devices. Section 3 (Results) analyzes the experiment that was followed in order to evaluate the proposed mechanism, whilst Section 4 (Discussion) describes the findings of our research, stating its innovative points. Finally, Section 5 (Conclusions) analyzes our conclusions that are accompanied with our plans for future research.

## 2. Materials and Methods

**Figure 1:**
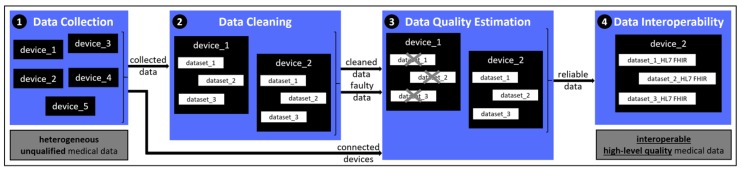
Architecture of the proposed mechanism.

The flowchart of the developed mechanism for achieving both data quality assessment and data interoperability is shown in Figure 1. More specifically, the mechanism consists of the stages of: (i) Data Collection, (ii) Data Cleaning, (iii) Data Quality Estimation, and (iv) Data Interoperability. 

### 2.1. Data Collection

**Figure 2:**
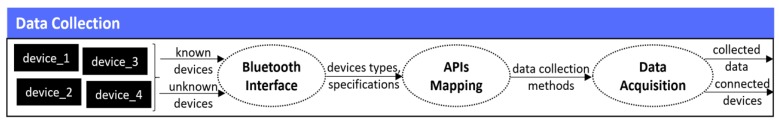
Data collection stage.

In the first stage of the mechanism, the discovery as well as the connection of the available heterogeneous IoT medical devices take place, followed by the collection of their data, as depicted in Figure 2. Therefore, the various available devices (e.g., activity trackers, body weight scales, etc.) are discovered and connected to the mechanism through the implementation of the approach proposed in our previous work in [21]. In more detail, this mechanism implements a Bluetooth interface, based upon the Bluetooth Low-Energy (BLE) [22], thus being able to communicate with devices that can be recognized and connected only via Bluetooth. As soon as the devices’ connections are successfully established, following the procedure of [21], the mechanism identifies all the Application Programming Interface (API) methods of the connected devices, extracting and using the ones that are responsible for collecting the data from the different connected devices. To this end, it should be mentioned that the mechanism is able to connect only devices that have open APIs in order to give access to their methods, since the private ones do not publicly offer information about their methods [23], whereas these devices might be either of known or of unknown nature (i.e., device type). Apart from this, it should be noted that since the IoT medical devices are separated into either medical grade or consumer grade devices [24], the proposed mechanism is able to detect and use both of them. 

### 2.2. Data Cleaning

**Figure 3:**
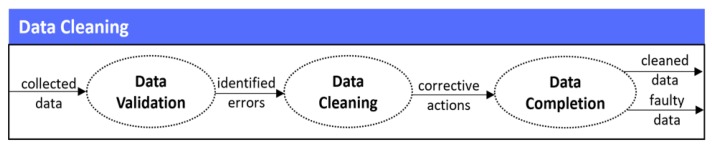
Data cleaning stage.

In the second stage of the mechanism, the cleaning of the data that has been collected takes place. In more detail, the developed data cleaning mechanism follows a multi-fold process that includes three (3) different steps, as depicted in Figure 3. 

More specifically, the steps are: (i) the data validation that identifies all the errors that are associated with conformance to specific constraints, safeguarding that the gathered data measures comply with defined business rules or constraints (i.e., conformance to specific data types, conformance to value representation, conformance to range constraints, conformance to pre-defined values, conformance to cross-field validity etc.), (ii) the data cleaning that eliminates the errors identified during the aforementioned validation process, by applying corrective actions upon the identified erroneous records of the data, and (iii) the data completion that handles the missing data, safeguarding that the provided data is fully complete and conforms to mandatory fields (i.e., required fields which cannot be empty). To this end, it should be noted that the identification of the errors and their cleaning (i.e., data validation and data cleaning steps) take into consideration well-defined rules that can be set by the IoT devices’ manufacturers, and do not necessarily employ advanced machine learning business logic in order to be performed. On the contrary, the handling of missing values (i.e., data completion step) is a significant research issue [25], and for this reason different mechanisms are employed according to the variation of the missing values. More specifically, in cases with low variation and frequent data samples, last non-zero values are used to substitute missing values, in cases with medium variation and frequent data samples, the moving average is used and the missing value is substituted with the average of the previous and the next non-zero values, while in cases with higher variation or with less frequent data samples, machine learning algorithms are employed to predict the missing values. To be more specific, the latter refers to: (a) the kNN imputation [26], which does not need to create an explicit predictive model to estimate the missing values, having the ability to easily treat examples with multiple missing values and both categorical and continuous variables, and (b) the C4.5 algorithm [27], which inherently ignores the missing values when calculating the features’ information, so that it can deal with multiple missing attributes in a dataset. 

Therefore, the Data Cleaning process takes into consideration the defined business rules, as well as the available current and historical data, in order to provide to the data completion step of the mechanism the cleaned, completed and properly validated data. Thus, the final cleaning results are produced, indicating the total corrective actions that were undertaken upon each different dataset, in combination with each derived cleaned dataset. In more detail, based on the obtained cleaning results, the accuracy (i.e., Data_Accuracy) of each different collected dataset is calculated following the Equation (1), where the Total_Records denote the number of the records that existed in each different dataset that derived from each different connected device, whereas the Total_Actions denote the number of the total errors (i.e., erroneous data, missing data, and dropped records) that were encountered during the whole cleaning process, in the corresponding datasets.

(1) $$\mathrm{Data}\_\mathrm{Accuracy}=\frac{\mathrm{Total}\_\mathrm{Records}\;–\;\mathrm{Total}\_\mathrm{Actions}}{\mathrm{Total}\_\mathrm{Records}}$$

Apart from this metric, based on the cleaning results, we calculate the number of the total faults (i.e., Faulty_Data) that existed in each different dataset that derived from each different connected device, following the Equation (2), where Data_Accuracy refers to the corresponding accuracy of each different dataset.

(2) Faulty_Data =1 − Data_Accuracy

Finally, we calculate the number of the completeness (i.e., Data_Completion) of each different dataset that derived from each different connected device, following the Equation (3), where the Total_Records denote the number of the records that existed in each different dataset, whereas the Dropped_Records denote the number of the records that were dropped (i.e., deleted) during the whole cleaning process, in the corresponding datasets.

(3) $$\mathrm{Data}\_\mathrm{Completion}=\frac{\mathrm{Total}\_\mathrm{Records}\;-\;\mathrm{Dropped}\_\mathrm{Records}}{\mathrm{Total}\_\mathrm{Records}}$$

### 2.3. Data Quality Estimation

**Figure 4:**
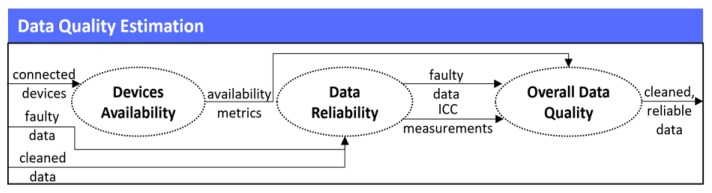
Data quality estimation stage.

In the third stage of the mechanism, the quality estimation of the connected devices’ cleaned data takes place. This stage is of major importance, since it is not sufficient to keep all the derived data and use it for further analysis, as much of it may have derived either from unreliable devices or from reliable devices that are faulty and error prone. For that reason, it is necessary to measure and evaluate the quality of all the produced data, so as to finally only keep the reliable data that comes from only reliable devices. In order to achieve that, as stated in our previous research in [28] for capturing the quality levels of data, it is more effective to estimate both the devices’ quality themselves, and the quality of their produced data. Thus, in this stage of the mechanism, three (3) different steps are followed, as they are illustrated in Figure 4.

#### 2.3.1. Devices Availability

In the first step of the Data Quality Estimation, the Devices Availability takes place, where the mechanism calculates the devices’ quality levels. Even though the research in [28] outlines that there exist a wide range of metrics for capturing the devices’ quality, in this mechanism, in order to calculate each different connected device’s quality, we measure only the metric of the availability (or mission capable rate) of them, as it is the most representative metric [29]. A wide range of availability classifications and definitions exist [30], however in this mechanism the most suitable one that is going to be measured is the operational availability. Operational availability (i.e., Availability) is the ratio of the system uptime to total time, given mathematically by the Equation (4), where the Operating_Cycle is the overall time period of operation being investigated and Uptime is the total time in which the system was functioning during the specific Operating_Cycle.

(4) $$\mathrm{Availability}=\frac{\mathrm{Uptime}}{\mathrm{Operating}\_\mathrm{Cycle}}$$

Therefore, we measure each device’s availability by getting the corresponding values, setting a timestamp in the developed mechanism in order to measure how often each device communicates with the latter and provides its data. 

#### 2.3.2. Data Reliability

However, as mentioned above, it is not sufficient enough to measure only the devices’ availability for deciding whether the latter is considered as qualitative or not, but it is more effective to measure also the quality (i.e., reliability) of these devices’ data. For that reason, we implement the second step of the Data Quality Estimation, where we use as an input from the Data Cleaning stage the number of the faulty data that derived upon each dataset, so as to correlate it with the availability results of the corresponding device that produced this dataset.

On top of this, in order to enhance the reliability of these results, in this stage we capture the reliability metrics [31] of each different dataset. More specifically, since in our case the reliability of the incoming data is measured upon the data that comes from the same patients, from the same types of devices, but in different periods of time, among the different types that exist for measuring data reliability [32], Test-Retest Reliability (TRR) is the most suitable one. Thus, based upon the fundamentals of TRR [33], in our case the measurements are taken by a single person (i.e., patient) on the same item (i.e., type of device), under the same conditions, and in a short period, evaluating the reliability across this period. In order to calculate the TRR of the connected devices’ data, the SPSS library [34] is used, calculating the corresponding Intraclass Correlation Coefficient (ICC), since the data may contain either interval or ratio data [35]. More specifically, we implement the method of two-way random effects, absolute agreement, and single rater/measurement (i.e., ICC (2,1)), obeying the corresponding conditions [33].

#### 2.3.3. Overall Data Quality

As a result, in the final step of the Data Quality Estimation, the Overall Data Quality occurs, where as soon as the ICC of each different dataset is calculated, its results are combined with the results of the availability (i.e., Availability) as well as the number of the faulty data (i.e., Faulty_Data) that derived upon the corresponding data, so as to finally decide whether each device, and as a result its derived data, are considered as of good quality or not. To this end, it should be noted that in order to consider the final results (i.e., Overall_Quality) as trustful and reliable, these must exceed the set threshold of 90%. In more detail, Overall_Quality is calculated mathematically by Equation (5), where it equals with the sum of the subtraction of the calculated Faulty_Data from the corresponding device’s Availability that is multiplied with a weight of 0.7, and the corresponding ICC of the data of this device that is multiplied with a weight of 0.3. With regards to the set weights, these were chosen based upon the research results that were acquired during relevant experiments that were performed in the past. These results revealed that the Faulty_Data in combination with the Availability should have a higher weight than this of the ICC, since they were considered to be more characteristic and decisive for the calculation of the Overall_Quality results. Consequently, based on the calculated results of the Overall_Quality, all the data that exceed the set threshold are kept to the mechanism to be made interoperable, whilst the ones that do not exceed the set threshold are discarded by it.

(5) Overall_Quality=((Availability− Faulty_Data) ∗ 0.7) + (ICC ∗ 0.3))

### 2.4. Data Interoperability

**Figure 5:**
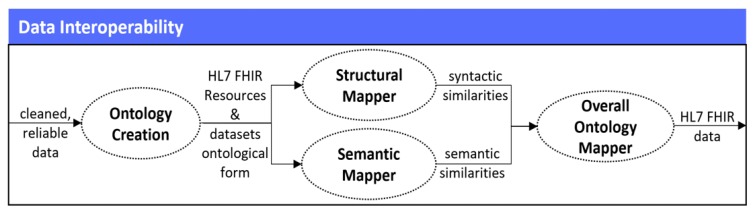
Data interoperability stage.

In the fourth stage of the mechanism, by having gathered only the data that is of high-levels of quality, its interpretation and transformation into the HL7 Fast Healthcare Interoperability (HL7 FHIR) standard [36] takes place, which is currently widely adopted among different healthcare organizations for achieving data interoperability [37]. That is why, an updated approach of the FHIR Ontology Mapper (FOM) that we proposed in [38] is implemented, having the ability to identify the similarities that exist between the different attributes of the HL7 FHIR resources and the attributes of the healthcare related datasets, and finally transform them into the HL7 FHIR format. In order to achieve that, the developed Data Interoperability mechanism follows four (4) different steps, as they are depicted in Figure 5.

#### 2.4.1. Ontology Creation

In the first step, the Ontology Creation takes place, which presents an automatic way for obtaining an initial organization of the ontological concepts from a collection of any documents that can be formed either by text or by structure and text. In our case, in order to create the ontologies, the implementation of the mechanism proposed in our work in [39] is followed. More particularly, all the collected datasets are preprocessed in order to identify the type of the document in which each dataset is stored (e.g., JSON, CSV, etc.), and converted into eXtensible Markup Language (XML) file format [40]. Following the 4-step transformation process of [39], each generated file is transformed into the corresponding ontologies, in the form of Resource Description Framework (RDF) entities [41]. In parallel, all the HL7 FHIR resources that exist in the FHIR resources’ list [36] are also structured in their ontological form, through the FHIR Linked Data Module [42].

#### 2.4.2. Structural Mapper

As soon as all the RDF entities (i.e., ontologies) are successfully constructed, the Structural Mapper takes place following our corresponding approach proposed in [38], thus providing a way for mapping and identifying the structural similarity between each collected dataset’s attributes and the HL7 FHIR resources’ attributes, based on their structural form (i.e., syntactic representation of their ontological names). Consequently, the goal of the Structural Mapper is to identify the similarity measure between the different ontological names, and provide the probability that a specific attribute of a dataset’s ontology is the same—in terms of its syntactic interpretation—with a specific attribute of the HL7 FHIR resources’ ontologies. Therefore, in our case, in order to measure the structural similarity between two (2) ontologies, the Structural Mapper automatically identifies and iterates over each different ontology that has been created from the previous step, and provides each ontology as an input to the developed mechanism. Afterwards, the structural representation of the names of the ontologies to their upper-case characters takes place, which are then split up into different character pairs (e.g., PATIENT is split up into {PA, TI, ENT}). Shortly, this part of the mechanism iterates over the structural form of each ontology between the names of the ontologies of the collected datasets and the names of the HL7 FHIR resources’ ontologies, and identifies word patterns—in terms of words that are frequently met and repeated. As soon as these patterns are identified, they are split and stored into different tables for each ontology, resulting into the identification of the different character pairs that are met. Sequentially, the next step deals with the checking of the multiple character pairs, in order to identify which characters can be found in both split strings. The outcome of this step is the total different character pairs that are similar between the ontologies that are compared to each other. Thus, in the final step, the calculation of the probability of the structural similarity of the aforementioned pairs occurs based on the structural similarity calculation equation provided to [38], storing finally the results into different tables. To be more precise, the probability of the structural similarity of the aforementioned pairs is calculated based on the equation presented in (6). Shortly, the structural similarity (i.e., St_Simil) between two (2) given ontologies S1 and S2, is twice the number of character pairs (i.e., Character_Pairs (S1), Character_Pairs (S2)) that are common to both names of the different ontologies, divided by the sum of the number of character pairs (i.e., Characters (S1), Characters (S2)) that are identified in both ontologies.

(6) $$\mathrm{St}\_\mathrm{Simil}=\frac{2\;*\;\left(\mathrm{Character}\_\mathrm{Pairs}\left(S1\right)\;\cap \;\mathrm{Character}\_\mathrm{Pairs}\left(S2\right)\right)}{\left(\mathrm{Characters}\left(S1\right)\;+\;\mathrm{Characters}\left(S2\right)\right)}$$

#### 2.4.3. Semantic Mapper

In the same concept of the Structural Mapper, the Semantic Mapper is implemented following our corresponding approach proposed in [38], thus providing the means for aligning and mapping the different ontologies of the collected datasets and the HL7 FHIR resources, according to their semantical meaning. Consequently, the Semantic Mapper’s whole process involves running several matching operations, according to the semantic similarity (i.e., relationships and dependencies between names’ structure and instances’ placement) among the ontologies that were constructed during the Ontology Creation, and then filtering their results so as to find an overall alignment. Hence, in our case, in order to measure the semantic similarity between the collected datasets and the HL7 FHIR resources’ ontologies, the identification of the different names, along with their relationships and instances takes place. As soon as the different triples of information are gathered, these are stored in different triples of arrays. In sequel, the comparison of these triples of arrays occurs, in order to calculate their semantic similarity. For that reason, the Retina API [43] is implemented in the form of a sparse distributed semantic space, which is based on the assumption that the language is stored in the human brain in the form of a distributed memory. In that case, the general English Retina Database [43] is being accessed, converting the different triples of arrays into semantic fingerprints in order to compare their meanings, by overlaying their semantic fingerprints and calculating their distance. By the time that all the different combinations of comparisons have occurred, different tables of the semantic similarity between the different triples of arrays are created. Hence, different probabilities of the correspondence between the attributes of the collected datasets’ ontologies and the attributes of the HL7 FHIR resources’ ontologies are calculated based upon the semantic similarity equation provided to [38], and stored into different tables. More specifically, as soon as the matching between the semantic meanings of the different ontologies has been performed, the metrics of the final results’ precision and recall are identified, in order to finally calculate the harmonic mean (i.e., Sem_Simil) of these two (2) measures. Thus, these results provide the number that indicates how much each specific ontology of the collected datasets is semantically the same with an ontology of the HL7 FHIR resources, with respect to the Unified Medical Language System (UMLS) reference alignment [44]. To calculate the Sem_Simil metric, based on [38], the metrics of both Precision and Recall must be calculated. Hence, sequentially, the Sem_Simil metric is calculated through Equation (7), which refers to the harmonic mean of the Precision and the Recall that have already calculated. It should be mentioned that, in essence, the Sem_Simil metric refers to the F_measure_ metric that is the traditional metric for capturing the harmonic mean of Precision and Recall.

(7) $$\mathrm{Sem}\_\mathrm{Simil}=\frac{2\;*\;\left(\mathrm{Precision}\;*\;\mathrm{Recall}\right)}{\left(\mathrm{Precision}\;+\;\mathrm{Recall}\right)}$$

#### 2.4.4. Overall Ontology Mapper

Based on the aforementioned results, the Overall Ontology Mapper takes place, which is the final step of the Data Interoperability stage. More specifically, the Overall Ontology Mapper provides a way for aggregating the results that have derived from the Structural Mapper and the Semantic Mapper, so as to identify the ontologies that have been properly mapped, and finally translate the attributes of these ontologies into the HL7 FHIR format. In our case, in order to calculate the aggregated final result, the Overall Ontology Mapper queries through the metrics and values that have been calculated for each different ontology, and provides the average (i.e., Mean) between the structural and the semantic similarities. Thus, this average is calculated as the total of the structural (i.e., St_Simil) and the semantic (i.e., Sem_Simil) similarities, divided by two, as stated in Equation (8).

(8) $$\mathrm{Mean}=\frac{\mathrm{St}\_\mathrm{Simil}\;+\;\mathrm{Sem}\_\mathrm{Simil}}{2}$$

Sequentially, according to the calculated mean of the structural and the semantic similarities, it is assumed that an attribute of a collected dataset is characterized that it matches to a specific attribute of the HL7 FHIR resources in the case that their mean is over the threshold of 90% (i.e., 90% structural and semantic similarity). In the case that the mean is lower than this threshold, then the attribute of the HL7 FHIR resource with higher probability of similarity with a specific attribute of the dataset, is automatically considered that it represents the specific attribute. The mechanism iterates for all the stored ontologies so as to finally identify the ontologies of the data and translate them into HL7 FHIR format. Therefore, through this way, the mechanism achieves to make all the collected reliable data interoperable, successfully transforming it into the HL7 FHIR format.

## 3. Results

### 3.1. Dataset Description

In order to perform a complete testing and evaluation of the proposed mechanism, twenty (20) IoT consumer grade medical devices were chosen, being able to communicate through Bluetooth with the mechanism. These devices have been selected since they represent different types of medical devices, offering open APIs for accessing their data. Some of these devices are of the same type, while being produced either by the same or by different manufacturers. To be more precise, for those twenty (20) chosen devices we had prior knowledge about their name, manufacturer, and device type (i.e., activity trackers (AT), blood pressure monitor (BPM), pulse oximeter (PO), body weight scale (BWS), and glucometer (GL)). As depicted in Table 1, three (3) of these devices were activity trackers, four (4) were blood pressure monitors, two (2) were pulse oximeters, nine (9) were body weight scales, and two (2) were glucometers.

### 3.2. Experimental Results

The proposed mechanism was developed in Java SE using the NetBeans IDE v8.0.2 [45], and used a processing environment with 16 GB RAM, Intel i7-4790 @ 3.60 GHz × 8 CPU Cores, 2 TB Storage, and Windows 10 operating system. Regarding the results of the mechanism, these are depicted below, following the four (4) stages described in Section 2 (Materials and Methods). It should be noted that the source code availability is not currently in open-source format, since it has not been finalized yet.

#### 3.2.1. Data Collection

**Figure 6:**
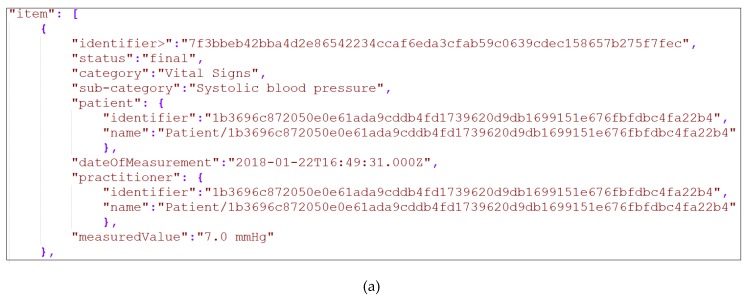

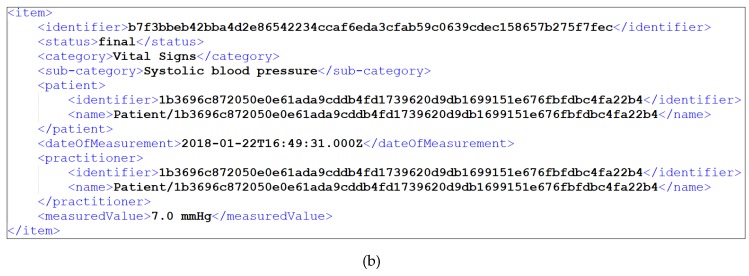
(**a**) Snapshot of Withings BPM data in JSON; (**b**) Snapshot of Withings BPM data in XML.

To begin our experiment, in the first stage of the mechanism (i.e., Data Collection) the chosen devices were used, where all these devices had to be connected to the mechanism. Following the procedure proposed in our work in [21], we identified all the API methods of the connected devices, extracting and using the ones that are responsible for collecting their medical data. Through this way, the mechanism gathered the data from all the connected devices. To this end, it should be noted that all the gathered data was in the form of JavaScript Object Notation (JSON) files, but for the purposes of the experiment it was automatically transformed into XML files via our mechanism. A snapshot of a JSON file and the corresponding XML file of one (1) of the chosen device’s (i.e., Withings BPM blood pressure monitor) produced data can be seen in Figure 6. What is more, it should be noted that the collected data were captured from the corresponding devices that were provided and used by an anonymized patient of BioAssist’s platform [46] who gave us her consent.

#### 3.2.2. Data Cleaning

Sequentially, in the second stage of the mechanism (i.e., Data Cleaning), the steps of the data cleaning process described in Section 2.2 (Data Cleaning) were applied, so as to clean all the collected data. Within the context of our experiment, the cleaning process was executed against the data that was collected over a period of two (2) months (i.e., 61 days), so as to facilitate the aggregation of enough data records. The cleaning process was applied across all the connected devices collected data. However, in the experimental results only the results of the cleaned data of the four (4) connected blood pressure monitors (i.e., iHealth Clear, iHealth Track, Withings BPM, and iHealth View) are described. Thus, for each one of the blood pressure monitors that we utilized, we triggered the Data Collection process four (4) times per day, even though in some cases slight differentiations occurred, resulting in slightly lower or slightly higher numbers of data records collected. Henceforth, concerning the total number of the records of the datasets that were received from the different blood pressure monitors into the 61 days, the iHealth Clear contained 238 records, the iHealth Track contained 239, the Withings BPM contained 244, and the iHealth View contained 243. It should be mentioned that since the data collection process was triggered four (4) times per day, under perfect conditions, each different blood pressure monitor should provide 244 records within these 61 days. It should be added that each one of these records contained three (3) different measurements, including the Diastolic Blood Pressure, the Systolic Blood Pressure, and the Heart Rate. All the records from all these devices contained the same elements (i.e., date and time of the observation, diastolic blood pressure (including its code, value, and unit), systolic blood pressure (including its code, value, and unit) and heart rate (including its code, value, and unit)). For each element, the corresponding constraints were defined, as they are presented in Table 2, whereas the snapshot of Figure 6 depicts an uncleaned observation of the Withings BPM blood pressure monitor.

It should be noted that during the cleaning process, for the required elements that were randomly missing, the corresponding records were discarded. For the non-required elements that were missing, the corresponding values were filled in with pre-defined values, whereas for the non-required elements missing at random, the machine learning techniques described in Section 2.2 (Data Cleaning) were employed. As a result, applying in all the acquired datasets the corresponding cleaning actions, the results of Table 3, Table 4, Table 5 and Table 6 were produced, demonstrating the cleaning actions that were applied upon the different attributes (i.e., Data-Time, Diastolic Blood Pressure Code/Value/Unit, Systolic Blood Pressure Code/Value/Unit, and Heart Rate Code/Value/Unit) of the four (4) connected blood pressure monitors (i.e., iHealth Clear, iHealth Track, Withings BPM, and iHealth View). In more detail, the columns of the Tables entitled as “Device Name” denote the name of each different connected blood pressure monitor. The columns entitled as “Erroneous Data” present the number of the records that the specific attribute did not conform to the validation rules documented in the first step (i.e., data validation) of the cleaning process. The columns entitled as “Corrective Actions” document the type of the actions taken for correcting the values of the erroneous attributes. The columns entitled as “Missing Data” signify the number of the records that their attributes contained missing values, whilst the columns entitled as “Corrective Actions” document the type of the actions taken for correcting the values of the missing attributes.

After the calculation of all the aforementioned results, the data cleaning results of each different connected blood pressure monitor were combined, resulting into the results of Table 7. More specifically, the column of the Table entitled as “Device Name” denotes the name of each different connected blood pressure monitor, the column entitled as “Initial Records” presents the total number of the records that were initially collected by each device, whilst the column entitled as “Final Records” presents the total number of the records that remained after the cleaning actions that were performed upon the datasets. Based on the results of Table 3, Table 4, Table 5 and Table 6, the column entitled as “Errors Encountered” denotes the total number of both the erroneous and the missing data values—including the records that were dropped, the column entitled as “Records Dropped” provides the total number of both the erroneous and the missing corrective actions that had dropped (i.e., deleted) records (i.e., triples of measurements), while the column entitled as “Errors Corrected” denotes the total number of both the erroneous and the missing data values—excluding the records that were dropped. The column entitled as “Data Accuracy” provides the percentage of the accuracy of the collected data that were cleaned. What is more, the column entitled as “Faulty Data” denotes the total number of the faulty data that derived upon the different datasets, which is one of the most important metrics of the Data Cleaning process of our mechanism, and is going to be used in the Data Quality Estimation stage. Finally, the column entitled as “Data Completion” denotes the final percentage of data completion after the successful cleaning of the data. To this end, it should be noted that in order to calculate the “Data Accuracy”, the “Faulty Data” as well as the “Data Completion” percentages, the corresponding equations of (1), (2), and (3) of Section 2.2 (Data Cleaning) were implemented.

**Figure 7:**
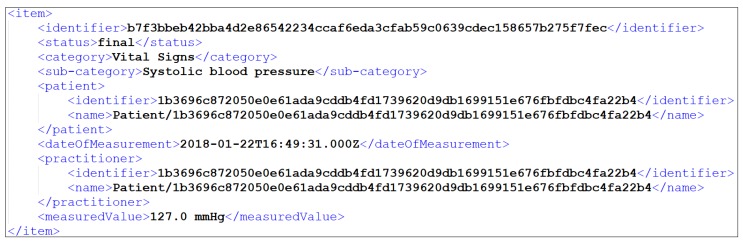
Snapshot of Withings BPM cleaned data in XML.

The same cleaning process was executed on the remaining sixteen (16) different collected datasets by applying the corresponding predefined constraints, thus implementing all the needed cleaning actions, and updating finally the corresponding XML files of the datasets. Following the snapshot of Figure 6, the depicted item contained a faulty value of systolic blood pressure (i.e., 7), which was corrected with the kNN imputation as depicted in Table 5. More specifically, the new corrected value was 127, as it is illustrated in the attribute “measuredValue” in Figure 7.

#### 3.2.3. Data Quality Estimation

In sequel, in the third stage of the mechanism (i.e., Data Quality Estimation), the calculation of the overall data quality of the devices’ cleaned datasets occurred, by combining (i) the availability of each device, (ii) the number of the faulty data that was calculated during the Data Cleaning process for the collected dataset of each corresponding device, and (iii) the ICC of this data, following the procedure described in Section 2.3 (Data Quality Estimation). Therefore, we captured the overall data quality of the twenty (20) connected devices by measuring all the aforementioned metrics. However, in the experimental results, following the example of the Data Cleaning process, only the results of the four (4) connected blood pressure monitors (i.e., iHealth Clear, iHealth Track, Withings BPM, and iHealth View) are described. 

More specifically, regarding the availability measurements of these devices, we measured their uptime through the frequency of their data transmission to the mechanism every day for the period of the 61 days. Therefore, we captured the availability for each one of these devices, assuming that a device is fully available (i.e., 100% availability) when it sends four (4) records (i.e., triples of measurements) per 24 hours (i.e., per day), resulting in 244 records for the total 61 days of the experiment. After iterating this process, we resulted into Table 8 that depicts the results that we collected, having performed the same experiment for 61 days in a row. In more detail, Table 8 summarizes our results including the column “Device Name” that denotes the name of each different connected blood pressure monitor, the column “Final Records” that denotes the total number of the records that remained after the cleaning of each device’s dataset, the column “Availability” that presents the percentage of the data availability of these devices, considering the data availability of the aforementioned fully available device, the column “Faulty Data” that presents the percentage of the faulty data that resulted from the Data Cleaning process, the column “ICC” that denotes the TRR of the devices’ collected data, and the column “Overall Quality” that presents the final percentage of the devices’ derived overall data quality. To this end, it should be noted that in order to calculate the “Overall Quality” the Equation (5) of Section 2.3 (Data Quality Estimation) was implemented.

As stated in Section 2.3 (Data Quality Estimation), in order to consider the final results (i.e., Overall Quality) of each device’s data as trustful and reliable, and thus keep it for making it interoperable, these must exceed the set threshold of 90%. Thus, based upon the results of Table 8, it can be observed that all the cleaned data was of high-levels of quality, as it exceeded the set threshold, whereas the Withings BPM had the best Overall Quality results among all the other blood pressure monitors (highlighted with gray). As a result, all the data of all the blood pressure monitors kept in the mechanism in order to become interoperable in the next stage of the mechanism. The same quality process was executed on the remaining sixteen (16) collected datasets, by capturing their overall quality (i.e., Overall Quality), and thus concluding whether these would be kept into the mechanism for further analysis or not, depending on whether their overall quality percentages exceeded the set threshold or not. It should be noted that in the case that a device’s overall quality did not exceed the set threshold, then this device was discarded by the mechanism, totally erasing its data from it.

#### 3.2.4. Data Interoperability

Finally, in the fourth stage of the mechanism (i.e., Data Interoperability), all the steps of the data interoperability process that were described in Section 2.4 (Data Interoperability) were employed, in order to transform the cleaned data of high-levels of quality into the HL7 FHIR format.

**Figure 8:**
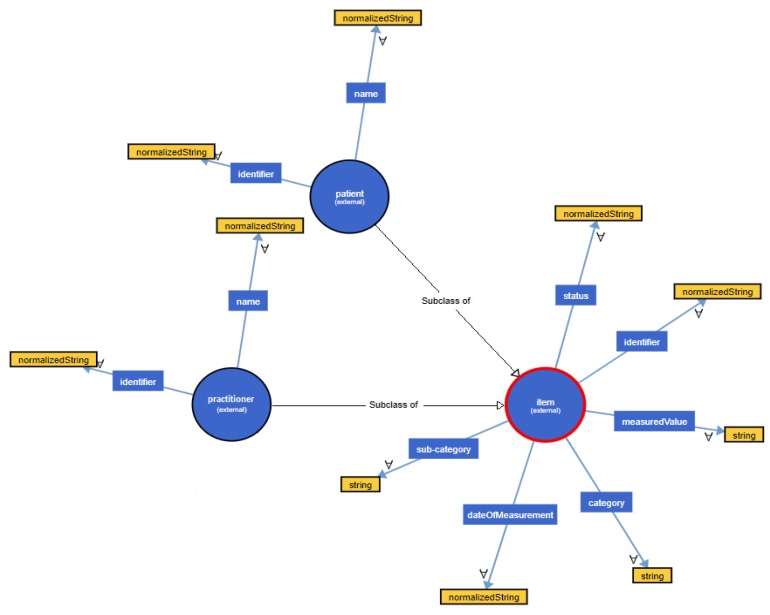
Ontological form of Withings BPM dataset.

Since the data was already in XML format, we did not have to transform it into another format. Therefore, our initial step was to transform the provided data into their ontological form. Based on the snapshot of the cleaned dataset that was provided by the Withings BPM (Figure 7), the ontological hierarchical tree that was created is depicted in Figure 8, visualizing the different names, relationships and instances of the constructed ontology. It should be noted that the same process is repeated for all the different datasets that were not discarded during the Data Quality Estimation process, in order to get their ontological form.

Sequentially, in the next step we identified the structural similarity that existed among the attributes of the different datasets and the HL7 FHIR resources, comparing the names of the corresponding constructed ontologies, following the process of the Structural Mapper described in Section 2.4 (Data Interoperability). As soon as the Structural Mapper performed on the different combinations, it provided the results of Table 9. Shortly, Table 9 depicts a snapshot of the top-2 structural similarities between the HL7 FHIR resources’ attributes and the attributes of the snapshot of the dataset that was provided by the Withings BPM (Figure 7).

In the next step of Data Interoperability, the identification of the semantic similarity that existed among the different constructed ontologies of the datasets and the HL7 FHIR resources occurred, taking into account the different names, relationships and instances that could have been identified. Consequently, following the process of the Semantic Mapper described in Section 2.4 (Data Interoperability), the latter performed on the different combinations, providing the results of Table 10. As in the previous step, Table 10 depicts a snapshot of the top-2 semantic similarities between the HL7 FHIR resources’ ontologies and the ontologies of the snapshot of the dataset that was provided by the Withings BPM (Figure 7).

Afterwards, as soon as the results of the Structural and the Semantic Mapper had been captured, the Overall Ontology Mapper took place, deriving the results of Table 11, which depicts the largest calculated pairs of means of the Structural and the Semantic Mapper results.

**Figure 9:**
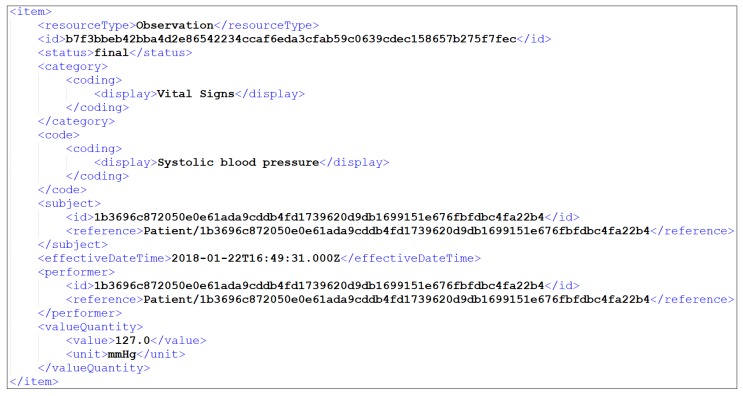
Snapshot of Withings BPM interoperable data in XML.

It should be noted that in Table 11, only the largest calculated pairs of means of the Structural and the Semantic Mapper results are depicted, since it would be almost impossible to illustrate all the derived combinations. For instance, in Table 9 it can be seen that the practitioner dataset attribute had greater value in structural similarity with the Practitioner HL7 FHIR resource (100.0%), while in Table 10 it can be seen that the same dataset attribute (i.e., practitioner) had greater value in semantic similarity with the Observation.subject HL7 FHIR resource (63.0%). However, after calculating all the pairs of means for the case of this specific similarity, we concluded that the greater value of the overall mean resulted into the fact that the practitioner dataset attribute was more similar with the Practitioner HL7 FHIR resource, concerning both the structural and the semantic similarity results. The same process was repeated for all the different pairs, concluding to the largest calculated pairs of means of the different similarities, and thus to the mapping of all the datasets to the HL7 FHIR format. Following the snapshot of Figure 7, the depicted item of Withings BPM was translated into HL7 FHIR format, as illustrated in Figure 9.

## 4. Discussion

The current research proposed a mechanism for assessing the quality of data that derive from heterogeneous IoT medical devices, and making it finally interoperable, following a 4-stepped approach. In order to evaluate this mechanism, we implemented a specific experiment, through which we tested the effectiveness of each different stage of the mechanism, focusing mainly on the three (3) key research stages of them, which were the Data Cleaning, the Data Quality Estimation, as well as the Data Interoperability stages.

Regarding the Data Cleaning process, as expected, several data records were found to be inherently “dirty”, either requiring the entire data record to be dropped, or requiring the data record to be completed by means of filling in missing values. Nevertheless, after the implementation of the Data Cleaning process, the completion of the data records increased significantly, as signified by the column “Data Completion” of Table 7. In order to achieve these results, various mechanisms were employed for filling in missing values, including: (i) filling in missing values with pre-defined values in the case of erroneous or missing units, (ii) handling missing values using the kNN imputation in the case of correcting erroneous values of specific attributes (namely dropping the erroneous values such as out of range values and substituting them with imputed ones), and (iii) filling in missing values using the C4.5 imputation, since missing values were statistically more than the erroneous values. Thus, taking into consideration all these different cases, the results of the Data Cleaning process were of high accuracy. 

With regards to the Data Quality Estimation process, as mentioned in Section 2.3 (Data Quality Estimation), it needed as a prerequisite for its successful implementation the Data Cleaning results, referring to the faulty data that had derived from it. Based on the captured results of Table 8, we can conclude that through this process it is effective to decide whether each device’s derived data is considered as reliable or not, by combining: (i) the results of the availability, (ii) the number of the faulty data that derived upon the different data, as well as (iii) the corresponding reliability of this data based upon its ICC measurements. More specifically, as it can be observed, all the collected data that was cleaned was finally considered to be of high-quality, as it exceeded the set threshold. Since the threshold was set in 90%—a very demanding value, this indicated that the devices’ derived data was of high-quality, a fact that was verified from the quality measurements (i.e., availability, faulty data, reliability) that were manually calculated upon this data.

In deeper detail, Table 12 depicts the manually captured quality results (i.e., Manual Results) of the four (4) connected blood pressure monitors, in combination with the corresponding proposed mechanism’s quality results (i.e., Automatic Results). As it can be observed in Table 12, the iHealth Track’s manually calculated percentage of availability (highlighted with blue), has small differences (i.e., 0.41%) with the corresponding percentage of the mechanism (highlighted with yellow), without however affecting the overall results. This is because the mechanism accidentally discarded a record that it should not have been discarded, affecting the total number of records that were considered. Concerning the total number of the faulty data that were manually derived upon the different datasets (highlighted with blue), it can be observed that in some devices (i.e., iHealth Track, iHealth View), slight differences (i.e., 0.83% and 0.82% accordingly) were encountered compared with the mechanism’s results (highlighted with yellow). This is due to the fact that during the manual cleaning of the corresponding datasets, it was observed that the mechanism corrected a number of values that should not have been corrected, since the initial values were already correct. Nevertheless, with regards to the rest of the values that were corrected, the correction actions of the mechanism were successfully performed and adjusted. Concerning each device’s data reliability percentages of ICC, since the mechanism used the SPSS library for calculating them, and in the manual results we used the SPSS tool as well, the calculated results were identical. Therefore, since the calculation of the Overall Quality depended on all of the aforementioned metrics, its manually calculated final values were different as well, due to the differences in the highlighted cells of Table 12. Henceforth, since there were found inconsistencies only upon the measurements of the iHealth Track and iHealth View devices, their final overall quality results were differentiating as well. 

**Figure 10:**
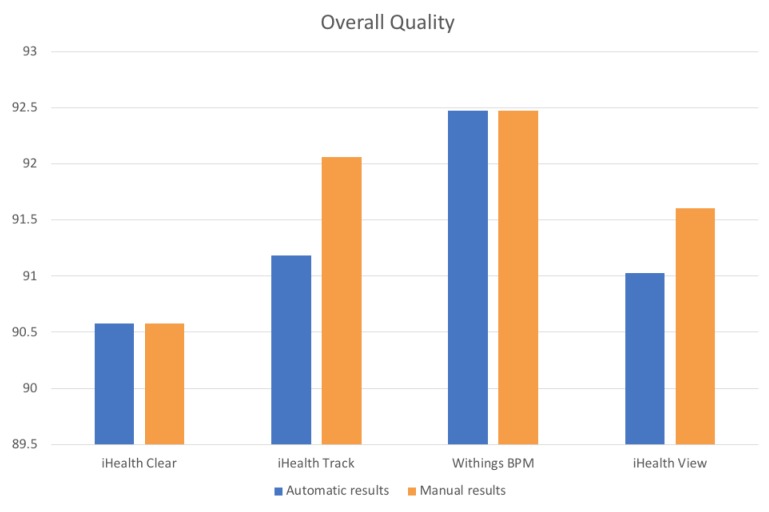
Comparison between manual and automatic overall quality results.

Figure 10 visualizes the results of Table 12, depicting the percentages of the manually and the mechanism’s calculated results, regarding the overall quality of the four (4) blood pressure monitors.

However, as it can be observed in Figure 10, the differences between the manual and the automatic results were not remarkable, since the absolute difference of their overall quality percentages was extremely small (approximately 0.05%), with the Withings BPM device having again the best quality results. Consequently, it becomes clear that the proposed mechanism provided highly effective and reliable results, concerning the devices overall quality. However, in some cases, like the one of the Withings BPM, we conclude that it is not always proper to derive rules or patterns about the overall quality of the data, setting the same weights of necessity for both the availability and the faulty data. More specifically, as it can be observed in Table 12, despite the fact that the Withings BPM transmitted data in a regular basis, achieving almost 100% availability, the latter contained a relatively large number of errors/faults (i.e., 8.20), slightly reducing its overall quality (i.e., 92.47%) that still remained in high-levels. On the contrary, the number of the faulty data should have a more significant impact in the final result of the overall quality, as it is more effective to produce and send reliable-unfaulty data, instead of sending data in a basis of 100% availability. For that reason, additional experiments should take place to obtain a more global view of the overall quality of the data, implementing different degrees of weights among the availability and the faulty data measurements.

Regarding the Data Interoperability process, it is clear that in order to identify and map ontologies, both structural and semantical mappings have to be applied. In more detail, through Table 9, Table 10 and Table 11, it can be observed that despite the fact that a dataset attribute has been mapped with a specific HL7 FHIR resource attribute due to their structural similarity, the same dataset attribute has been also mapped to a different HL7 FHIR resource attribute due to their semantic similarity, at the same time. Thus, there might be cases that an ontology may have structural similarity with a specific HL7 FHIR resource ontology (e.g., Patient.name dataset attribute had 100% structural match with the Patient.name FHIR resource attribute), but it may not have any semantic similarity with this specific HL7 FHIR resource ontology (e.g., Patient.name dataset attribute had 47% semantical mapping with the Patient.name FHIR resource attribute, whereas it had the greatest semantic similarity with Observation.subject.name FHIR resource attribute). Consequently, we are not able to create patterns and rules mentioning that in the case that an ontology matches structurally or semantically with a specific ontology, it will have always an exact match with it. 

However, the overall mechanism provides reliable results, since all the provided results have been also calculated manually and compared with the aforementioned results. For that purpose, the specific dataset’s attributes were mapped manually with the HL7 FHIR resources’ attributes to compare the results of the proposed Data Interoperability process with the actual outcomes. This was the main reason that a small sample of attributes was chosen for the process evaluation, in order to manually conclude more easily to these results. Table 13 illustrates the results of the manual transformation (i.e., Manual Results), in comparison with the automatic results of the proposed mechanism (i.e., Automatic Results). Based on the results of Table 13, it can be observed that there exist cases where the results of the developed process were not efficient, such as in the case of the Observation.effectiveDateTime, where the automated transformation provided results of low value, which however were identical with the results of the manual transformation. Hence, we can conclude that the Data Interoperability process provided results of 100% accuracy. 

**Figure 11:**
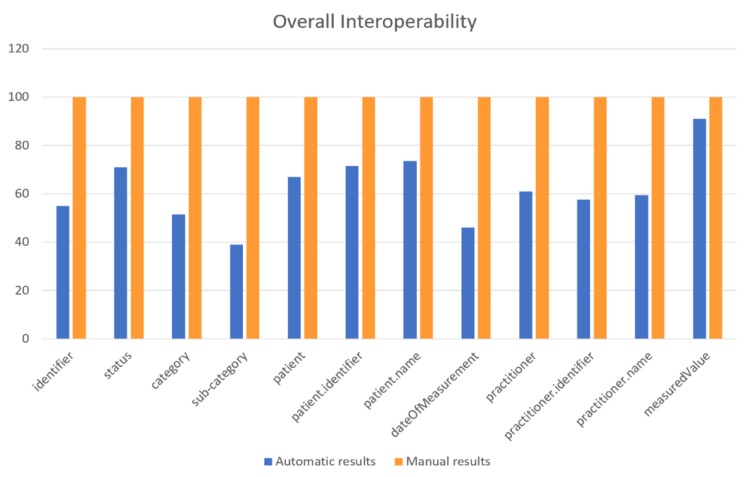
Comparison between manual and automatic overall interoperability results.

Figure 11 visualizes the results of Table 13, depicting the percentages of the manually as well as the mechanism’s calculated results, regarding the overall interoperability, based upon the cleaned and reliable data of the four (4) 4 blood pressure monitors.

## 5. Conclusions

It is an undeniable fact that devices’ data management is a very demanding research topic in the IoT area, characterized by a plethora of challenges that are currently far from solved. One of these challenges is the one of gathering heterogeneous IoT medical devices’ data, extracting the data that is of high-quality, and thus transforming only this data into a common format, ignoring the ones that are of low-level quality. For that reason, in this manuscript we have studied the challenging topics of both data quality and data interoperability, considering data that is coming from heterogeneous IoT medical devices of both known and unknown nature. By implementing this solution, we achieved to develop an innovative approach that can be interoperable and pluggable to different IoT platforms, regardless of the nature and the format of the data that they can manipulate.

More particularly, through our approach a mechanism of four (4) stages was implemented for coping exactly with this challenge. Based on this mechanism, initially all the available heterogeneous IoT medical devices were discovered and connected into the mechanism, in order to collect their data. Once the data was gathered, its cleaning took place by identifying and eliminating possible errors that encountered, whereas completing possible missing values, thus safeguarding that the provided data was fully complete and conformed to required attributes. Sequentially, these results were combined with the overall data quality measurements that were captured from the different devices, so as to decide whether each connected devices’ derived data would be considered as reliable or not, and as a result it would be kept in order to be translated into an interoperable format, and used for further analysis. Thus, finally the transformation of all the acquired reliable data into a common format occurred. The aforementioned mechanism was evaluated through a specific experiment, concluding that it was sufficient enough for assessing heterogeneous IoT medical devices’ data quality and interoperability.

Our future work includes that the mechanism will be tested with a huge amount of multiple heterogeneous IoT medical devices of different types. Apart from this, we aim to extend the list of the Data Cleaning already supported techniques, by implementing additional techniques such as the moving average method, and the interpolation and extrapolation methods. Moreover, we are willing to extend the Data Cleaning process by setting personalized constraints and rules that the collected medical data have to obey, considering that this data may come from different patients that may have different health statuses. On top of this, in the Data Quality Estimation process we aim to create a registry that will store in separate sub-registries the specifications (e.g., name, manufacturer, etc.) of the devices for whom the data has been considered as reliable. Consequently, the devices for which we will need in the future to identify their derived data quality will be compared with the aforementioned registries, and in the case that they will have some common specifications with the already stored devices’ specifications, they will be considered as of high-quality, bypassing the step of the devices’ availability estimation. Furthermore, with regards to the Data Interoperability process, we are planning to construct an automated tool that would implement different ontology matching techniques, since having a formal representation of these matchings will be useful for extending our mechanism. Furthermore, we will continue evaluating the proposed Data Interoperability process with multiple datasets of various medical standards and formats. It should be noted that in the cases that the underlying physical mechanism of capturing the physiological data is different among the tested devices, regarding the Data Interoperability process, the mechanism does not take into account the values of the gathered data, as it only deals with the format of the data. However, regarding the Data Cleaning and the Data Quality Estimation processes, the proposed mechanism will be updated in order to identify these cases, and will either inform the end user to terminate the overall process or ignore it in the case that this difference in the gathered data is not causing anomalies of vital importance. Finally, we aim to implement a visualization module providing detailed information about the connected devices and their derived data. More particularly, through this interface, the end users will be able to observe and manage their connected devices, being able to monitor the state and the location of their devices, visualize the acquired data, whereas observing all the transformations that the data is subjected to, being able to finally store all the transformed data.

## Figures and Tables

**Table 1:** Devices’ specifications.

#	Name	Manufacturer	Type	#	Name	Manufacturer	Type
***1***	iHealth Clear	iHealth	BPM	***11***	iHealth View	iHealth	BPM
***2***	Fitbit Aria 2	Fitbit	BWS	***12***	Withings BPM	Withings	BPM
***3***	iHealth Lite	iHealth	BWS	***13***	Polar Balance	Polar	BWS
***4***	iHealth Air	iHealth	PO	***14***	iHealth Smart	iHealth	GL
***5***	Withings Body	Withings	BWS	***15***	Withings Body Cardio	Withings	BWS
***6***	iHealth Track	iHealth	BPM	***16***	iHealth Wave	iHealth	AT
***7***	Garmin Index Smart Scale	Garmin	BWS	***17***	Xiaomi Mi Body Composition Scale	Xiaomi	BWS
***8***	Withings Steel	Withings	AT	***18***	NONIN 3230	NONIN	PO
***9***	Withings Body+	Withings	BWS	***19***	Garmin Vivofit	Garmin	AT
***10***	iHealth Align	iHealth	GL	***20***	iHealth Core	iHealth	BWS

**Table 2:** Elements’ constraints.

Element Name	Data Type	Required	Constraints
Date	String	Yes	Format: YYYY-MM-DD
Time	String	Yes	Format: HH:MM:SS
Diastolic Blood Pressure Code	UUID	Yes	Unique Identifier
Diastolic Blood Pressure Value	Integer	No	Range Constraints
Diastolic Blood Pressure Unit	String	No	mmHg
Systolic Blood Pressure Code	UUID	Yes	Unique Identifier
Systolic Blood Pressure Value	Integer	No	Range Constraints
Systolic Blood Pressure Unit	String	No	mmHg
Heart Rate Code	UUID	Yes	Unique Identifier
Heart Rate Value	Integer	No	Range Constraints
Heart Rate Unit	String	No	mmHg

**Table 3:** Date-Time cleaning results.

Device Name	Erroneous Data	Corrective Actions	Missing Data	Corrective Actions
iHealth Clear	0	None	0	None
iHealth Track	0	None	1	Dropped record
Withings BPM	0	None	0	None
iHealth View	1	Dropped record	2	Dropped record

**Table 4:** Diastolic Blood Pressure cleaning results.

Device Name	Erroneous Data	Corrective Actions	Missing Data	Corrective Actions
**Diastolic Blood Pressure Code**				
iHealth Clear	0	None	0	None
iHealth Track	0	None	0	None
Withings BPM	0	None	0	None
iHealth View	0	None	0	None
**Diastolic Blood Pressure Value**				
iHealth Clear	2	kNN imputation	3	C4.5 imputation
iHealth Track	1	kNN imputation	2	C4.5 imputation
Withings BPM	2	kNN imputation	4	C4.5 imputation
iHealth View	3	kNN imputation	3	C4.5 imputation
**Diastolic Blood Pressure Unit**				
iHealth Clear	0	None	0	None
iHealth Track	0	None	0	None
Withings BPM	0	None	0	None
iHealth View	0	None	0	None

**Table 5:** Systolic Blood Pressure cleaning results.

Device Name	Erroneous Data	Corrective Actions	Missing Data	Corrective Actions
**Systolic Blood Pressure Code**				
iHealth Clear	0	None	0	None
iHealth Track	0	None	0	None
Withings BPM	0	None	0	None
iHealth View	0	None	1	Dropped record
**Systolic Blood Pressure Value**				
iHealth Clear	2	kNN imputation	2	C4.5 imputation
iHealth Track	2	kNN imputation	1	C4.5 imputation
Withings BPM	1	kNN imputation	4	C4.5 imputation
iHealth View	1	kNN imputation	3	C4.5 imputation
**Systolic Blood Pressure Unit**				
iHealth Clear	1	Filled with value	1	Filled with value
iHealth Track	1	Filled with value	0	None
Withings BPM	1	Filled with value	2	Filled with value
iHealth View	0	None	0	None

**Table 6:** Heart Rate cleaning results.

Device Name	Erroneous Data	Corrective Actions	Missing Data	Corrective Actions
**Heart Rate Code**				
iHealth Clear	0	None	0	None
iHealth Track	1	Dropped record	0	None
Withings BPM	0	None	1	Dropped record
iHealth View	0	None	0	None
**Heart Rate Value**				
iHealth Clear	3	kNN imputation	3	C4.5 imputation
iHealth Track	2	kNN imputation	2	C4.5 imputation
Withings BPM	1	kNN imputation	4	C4.5 imputation
iHealth View	3	kNN imputation	5	C4.5 imputation
**Heart Rate Unit**				
iHealth Clear	0	None	0	None
iHealth Track	0	None	0	None
Withings BPM	0	None	0	None
iHealth View	0	None	0	None

**Table 7:** Overall cleaning results.

Device Name	Initial Records	Final Records	Errors Encountered	Records Dropped	Errors Corrected	Data Accuracy (%)	Faulty Data (%)	Data Completion (%)
iHealth Clear	238	238	17	0	17	92.86	7.14	100.00
iHealth Track	239	237	13	2	11	94.56	5.44	99.16
Withings BPM	244	243	20	1	19	91.80	8.20	99.59
iHealth View	243	239	22	4	18	90.95	9.05	98.35

**Table 8:** Overall quality results.

Device Name	Cleaned Records	Availability (%)	Faulty Data (%)	ICC (%)	Overall Quality (%)
iHealth Clear	238	97.54	7.14	91.00	90.58
iHealth Track	237	97.13	5.44	90.00	91.18
Withings BPM	243	99.59	8.20	95.00	92.47
iHealth View	239	97.95	9.05	96.00	91.03

**Table 9:** Structural similarity between attributes of HL7 FHIR resources and Withings BPM dataset.

Dataset Attribute	Top-1 Similarity		Top-2 Similarity	
	HL7 FHIR Resource Attribute	Structural Similarity (%)	HL7 FHIR Resource Attribute	Structural Similarity (%)
identifier	Observation.identifier	54	DiagnosticReport.identifier	23
status	Observation.status	61	DiagnosticReport.status	34
category	Observation.category	29	DiagnosticReport.category	22
sub-category	Observation.category	34	DiagnosticReport.category	28
patient	Patient	100	-	0
patient.identifier	Patient.identifier	100	-	0
patient.name	Patient.name	100	-	0
dateOfMeasurement	Observation.effectiveDateTime	56	Observation.valueDateTime	32
practitioner	Practitioner	100	-	0
practitioner.identifier	Practitioner.identifier	100	-	0
practitioner.name	Practitioner.name	100	-	0
measuredValue	Observation.valueQuantity	82	Observation.valueRange	13

**Table 10:** Semantic similarity between attributes of HL7 FHIR Resources and Withings BPM dataset.

Dataset Attribute	Top-1 Similarity		Top-2 Similarity	
	HL7 FHIR Resource Attribute	Semantic Similarity (%)	HL7 FHIR Resource Attribute	Semantic Similarity (%)
identifier	Observation.identifier	56	Patient.identifier	39
status	Observation.status	81	DiagnosticReport.status	17
category	Observation.category	74	DiagnosticReport.category	21
sub-category	Observation.code	44	Observation.category	32
patient	Observation.subject	39	Patient	34
patient.identifier	Observation.subject.identifier	54	Patient.identifier	43
patient.name	Observation.subject.name	53	Patient.name	47
dateOfMeasurement	DiagnosticReport. effectiveDateTime	53	Observation. effectiveDateTime	36
practitioner	Observation.subject	63	Practitioner	22
practitioner.identifier	Observation.subject.identifier	68	Practitioner.identifier	15
practitioner.name	Observation.subject.name	74	Practitioner.name	19
measuredValue	Observation.valueQuantity	100	-	0

**Table 11:** Overall similarity results Withings BPM dataset.

Dataset Attribute	HL7 FHIR Resource Attribute	Overall Similarity (%)
identifier	Observation.identifier	55.0
status	Observation.status	71.0
category	Observation.category	51.5
sub-category	Observation.code	39.0
patient	Patient	67.0
patient.identifier	Patient.identifier	71.5
patient.name	Patient.name	73.5
dateOfMeasurement	Observation.effectiveDateTime	46.0
practitioner	Practitioner	61.0
practitioner.identifier	Practitioner.identifier	57.5
practitioner.name	Practitioner.name	59.5
measuredValue	Observation.valueQuantity	91.0

**Table 12:** Manual and automatic overall quality results.

Device Name	Availability (%)	Faulty Data (%)	ICC (%)	Overall Quality (%)
**Manual Results**				
iHealth Clear	97.54	7.14	91.00	90.58
iHealth Track	97.54	4.60	90.00	92.05
Withings BPM	99.59	8.20	95.00	92.47
iHealth View	97.95	8.23	96.00	91.60
**Automatic Results**				
iHealth Clear	97.54	7.14	91.00	90.58
iHealth Track	97.13	5.44	90.00	91.18
Withings BPM	99.59	8.20	95.00	92.47
iHealth View	97.95	9.05	96.00	91.03

**Table 13:** Manual and automatic overall ontology mapper results.

Method Identifier	Manual Results	Similarity (%)	Automatic Results	Similarity (%)
identifier	Observation.identifier	100	Observation.identifier	55.0
status	Observation.status	100	Observation.status	71.0
category	Observation.category	100	Observation.category	51.5
sub-category	Observation.code	100	Observation.code	39.0
patient	Observation.subject (subject is Patient)	100	Patient	67.0
patient.identifier	Observation.subject.identifier (subject is Patient)	100	Patient.identifier	71.5
patient.name	Observation.subject.name (subject is Patient)	100	Patient.name	73.5
dateOfMeasurement	Observation.effectiveDateTime	100	Observation.effectiveDateTime	46.0
practitioner	Observation.subject (subject is Practitioner)	100	Practitioner	61.0
practitioner.identifier	Observation.subject.identifier (subject is Practitioner)	100	Practitioner.identifier	57.5
practitioner.name	Observation.subject.name (subject is Practitioner)	100	Practitioner.name	59.5
measuredValue	Observation.valueQuantity	100	Observation.valueQuantity	91.0
